# Peripheral blood eosinophil and classification of residual hematoma help predict the recurrence of chronic subdural hematoma after initial surgery

**DOI:** 10.3389/fsurg.2022.970468

**Published:** 2022-09-23

**Authors:** Sichao Chen, Linqian Shao, Li Ma

**Affiliations:** Department of Neurosurgery, Sir Run Run Shaw Hospital, Zhejiang University School of Medicine, Hangzhou, China

**Keywords:** chronic subdural hematoma, recurrence, burr hole, computerized tomography, eosinophil

## Abstract

Chronic subdural hematoma (CSDH) is a common type of intracranial hemorrhage in neurosurgical practice, whose incidence has increased markedly in recent years. However, CSDH still troubles clinicians with a high postoperative recurrence rate. The presence of eosinophils has been confirmed in some CSDH surgical specimens. Furthermore, postoperative residual hematoma has not been classified, and its association with the recurrence of CSDH remains unknown. The present study aimed to test the hypothesis that the peripheral blood eosinophils and the classification of postoperative residual hematoma are significant for the prediction of CSDH recurrence after burr-hole surgery. A retrospective review of 258 CSDH patients who received burr-hole surgery was performed. A complete blood picture with differential count was taken before surgery. Clinical, laboratory, and radiographic factors predicting CSDH recurrence were identified in univariable and multivariable analyses. Univariable analysis showed that the percentage of eosinophils, peripheral blood eosinophil count <0.15 × 10^9^/L, gradation and separated types, postoperative volume of the residual cavity ≥70 ml, residual air volume ≥28 ml, residual hematoma volume ≥55 ml, residual hematoma width ≥1.4 cm, postoperative midline shift ≥5.6 mm, postoperative brain re-expansion rate <41%, postoperative low-density type, and postoperative high-density type correlated with the recurrence of CSDH. Multivariable analysis, however, showed that peripheral blood eosinophil count <0.15 × 10^9^/L, gradation and separated types, preoperative midline shift ≥9.5 mm, postoperative brain re-expansion rate <41%, postoperative low-density type, and postoperative high-density type are independent predictors for the recurrence of CSDH. We expect that peripheral blood eosinophils and the classification of postoperative residual hematoma may facilitate our understanding of the recurrence of CSDH after initial surgery.

## Introduction

Chronic subdural hematoma (CSDH) is one of the most common types of intracranial hemorrhage in neurosurgical practice, particularly among the elderly population (>65 years). The incidence of CSDH was reported to range widely from 1.72 to 20.6 per 100,000 persons per year (1), with an incidence that has increased markedly in recent years because of an aging society (2, 3). For asymptomatic patients, we tend to treat conservatively (4). When hematoma mass effect results in clinical symptomatology, such as headaches, seizures, and gait disturbance, surgical evacuation of the hematoma are preferred (4, 5). Surgical methods for evacuation include burr-hole, twist drill, and craniotomy (6, 7). Compared with the other two methods, burr-hole irrigation is the most widely used technique to treat CSDH with a lower recurrence rate and morbidity rate (8, 9). Even so, CSDH still troubles clinicians with a high postoperative recurrence rate ranging from 2.5% to 33% (10, 11).

Several studies have reported numerous potential risk factors associated with the recurrence of CSDH, including blood type A, older age, higher body mass index (BMI), bilateral operation, preoperative hematoma volume, postoperative hematoma residual cavity volume, computed tomography (CT) density, antiplatelet or anticoagulant use, midline shift, and some technical aspects of surgery (3, 7, 10–14). According to the internal architecture and density of each hematoma, CSDHs were classified into five types by Nakaguchi et al., including homogeneous (homogeneous hypodense, homogeneous isodense, and homogeneous hyperdense), gradation, laminar, separated, and trabecular types (15). It has recently been reported that these preoperative CT scan features be used to predict the risk of postoperative recurrence (10, 16). However, postoperative residual hematoma (PRH) has not been classified and its association with recurrence of CSDH has not been investigated to date and remains unknown. In some CSDH cases, the PRH can be classified into three types according to CT imaging within 48–72 h of surgery. We hypothesize that the classification of PRH may be useful for predicting the risk of postoperative recurrence.

CSDH is an inflammatory and angiogenic disease induced by trauma or other reasons (4). In addition to the increased concentration of inflammatory factors found in CSDH fluid, the presence of eosinophils within the outer membrane of CSDH has been confirmed in up to 50% of all CSDH surgical specimens (17, 18). Some studies suggest that eosinophils may promote hyperfibrinolysis within CSDH outer membranes and contribute to the growth of CSDH (17, 19). However, eosinophils have also been pointed out to play a protective role in the repair and healing process of these membranes (18, 20, 21). In addition, the presence of a dense eosinophilic infiltrate within CSDH outer membranes may be associated with a lower risk of recurrence (21). However, to date, the relationship between human peripheral blood eosinophils and CSDH has not been studied and remains unknown. Therefore, the present study aimed to retrospectively investigate the association of PRH and preoperative peripheral blood eosinophil count with the recurrence of CSDH after surgery.

## Methods

### Patients and definition of CSDH recurrence

In this retrospective cohort study, we investigated 258 patients who had been admitted to Sir Run Run Shaw Hospital for the burr-hole surgical treatment of CSDH between January 2015 and September 2020. All patients were diagnosed as CSDH using head CT scans before the operation and confirmed by operative findings. All patients underwent single burr-hole surgery under general anesthesia. Hematoma evacuation was performed by a single burr-hole and the hematoma cavity was irrigated with normal saline until the flushing fluid became clear. Finally, the hematoma cavity was connected to a closed drainage system for 48–72 h. Postoperative recurrence is defined as a symptomatic ipsilateral enlargement in the hematoma volume that required reoperation, which developed within 3 months of the initial surgery and was confirmed by the CT scan signs. The clinical outcome was the recurrence of CSDH. The patients routinely underwent repeated CT scans within 48–72 h postoperatively.

This study was approved by the Institutional Review Board of Sir Run Run Shaw Hospital for the study of human subjects. Due to the retrospective nature of the study, the requirement for informed consent was therefore waived.

### Demographic and clinical variables

The following demographic and clinical variables collected were included in the statistical analysis: age, sex, BMI, hypertension, diabetes mellitus, heart disease, chronic kidney disease, neurosurgery history, speech deficiency related to CSDH, spontaneous intracranial hypotension syndrome, preoperative Glasgow Coma Scale, use of antithrombotic drugs, thrombocytopenia, and coagulopathy. Patients with a platelet count lower than 100 × 10^9^/µl were considered to have thrombocytopenia. Prothrombin time, prothrombin percentage activity, international normalized ratio, activated partial thromboplastin time, thrombin time, or fibrinogen exceeding the normal range are considered coagulopathy.

Complete blood cell analysis was required for all patients before surgery. We collected some laboratory variables, including white blood cell count, platelet count, the percentage of neutrophils, the percentage of eosinophils, the percentage of basophils, peripheral blood neutrophil count, peripheral blood eosinophil count, and peripheral blood basophils count.

### Radiological variables

All patients included in the study underwent head CT scans before surgery and within 48–72 h after surgery. All CT scans of 258 patients with CSDH were analyzed by two senior neurosurgeons who were blinded to all patients’ clinical conditions. The following radiological variables were identified: unilateral or bilateral hematoma, CT imaging of different types of CSDH, preoperative hematoma width, preoperative midline shift, preoperative hematoma volume (PreHV), postoperative volume of the residual cavity (PostRCV), residual air volume (PostRAV), residual hematoma volume (PostRHV), residual hematoma width, postoperative midline shift, and CT imaging of different types of residual hematoma. Since not all patients with bilateral CSDHs underwent surgery on both sides, the variable of unilateral or bilateral hematoma and the variable of unilateral or bilateral surgery are all analyzed. The distance from the septum pellucidum to the sagittal suture was calculated as midline shift. PreHV, PostRCV, PostRAV, and PostRHV were calculated by the ABC/2 method proposed in the previous study (22). Postoperative brain re-expansion rate was calculated as follows: (PreHV − PostRCV)/PreHV × 100 (%).

**Figure 1:**
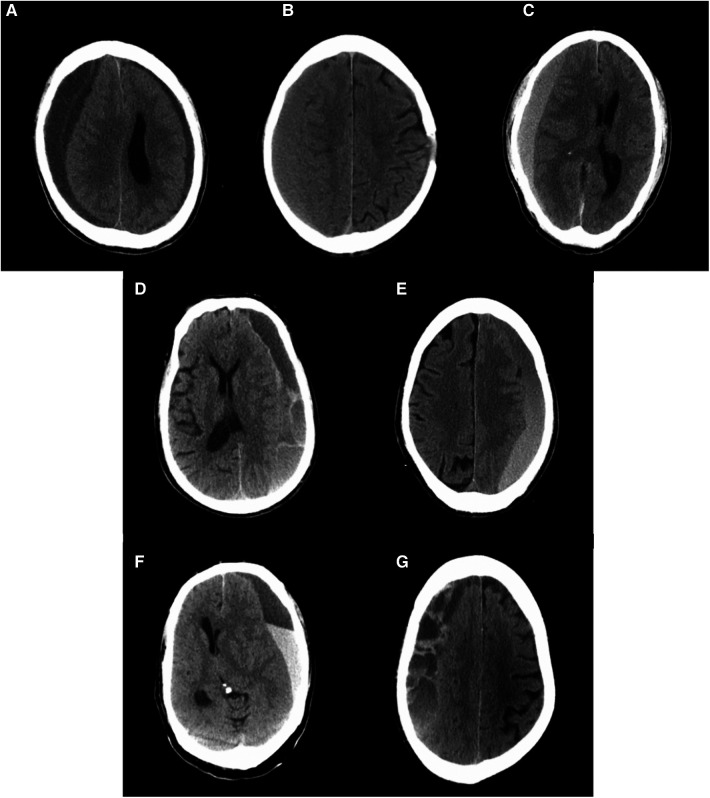
Computerized tomography scans of representative cases with chronic subdural hematomas according to their internal architectures described by Nakaguchi et al. (15). (**A**) Homogenous hypodense, (**B**) homogenous isodense, (**C**) homogenous hyperdense, (**D**) laminar type, (**E**) gradation type, (**F**) separated type, and (**G**) trabecular type.

**Figure 2:**
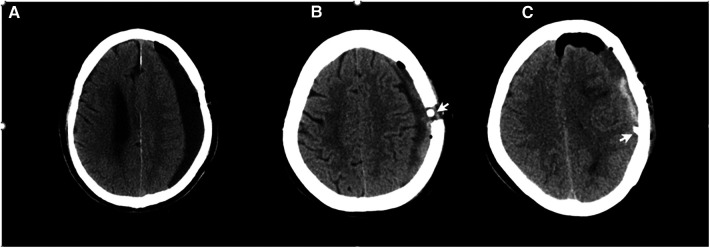
The classification of residual hematoma. The arrowhead points to the drain tube. (**A**) Postoperative hypo-density type, (**B**) postoperative hybrid-density, and (**C**) postoperative high-density type (see the explanation in the text).

CT imaging of CSDH was classified into five types according to the internal architecture and density of hematomas as described by Nakaguchi et al. and illustrated in Figure 1: (1) homogeneous type (homogeneous hypodense, homogeneous isodense, and homogeneous hyperdense), (2) separated type, (3) gradation type, (4) laminar type, and (5) trabecular type (10, 15). PRH was classified into three types using CT imaging within 48–72 h of surgery. The postoperative low-density type was defined as a residual hematoma exhibiting a homogenous low density similar to that of cerebrospinal fluid. The postoperative hybrid-density type was defined as a residual hematoma with a heterogeneous density, which is higher than that of cerebrospinal fluid, but not higher than that of brain parenchyma. If the residual hematoma is heterogeneous, with a higher density component than the brain parenchyma, this was called the postoperative high-density type (Figure 2).

### Statistical analysis

All statistical analyses were performed using SPSS for Windows, version 26.0 software (IBM Corp.). Discrete variables are expressed as number of patients (percentages). Continuous variables are described as the mean ± standard deviation (SD). The chi-square test for dichotomous variables and the independent sample *t*-test for continuous variables were used to assess the relationship between the investigated characteristics and CSDH postoperative recurrence. For intergroup comparisons, Fisher’s exact test, the chi-square test, or one-way ANOVA was used.

Receiver operating characteristic curve (ROC) with the calculation of area under the curve (AUC) was used to identify the cut-off values of continuous variables associated with CSDH recurrence. Logistic regression analyses were used to assess the predictors of CSDH recurrence. First, we used univariate logistic regression analyses to investigate the predictors of CSDH recurrence. Subsequently, variables that were significant at *p* < 0.10 in the univariable logistic regression were included in multivariate logistic regression analyses to test for an independent association with CSDH recurrence. The relation of each variable to CSDH recurrence is presented as an odds ratio (OR) and 95% confidence interval (CI). Values of *p* < 0.05 were used to indicate statistical significance.

## Results

During the study period, a total of 258 consecutive patients with CSDH who underwent burr-hole surgery were enrolled in this study. Thirty-seven patients (14.3%) were diagnosed with CSDH recurrence within 3 months from the initial burr-hole surgery for CSDH. The patients’ mean age was 69.86 ± 10.85 years with a range of 23–92 years. There were 214 men (82.9%) in this study group. Bilateral CSDH occurred in 70 (27.1%) patients, of whom 35 (13.6%) received bilateral surgery. The mean PreHV was 120.32 ± 40.51 ml, and the mean PostRCV was 69.22 ± 38.57 ml. The mean postoperative brain re-expansion rate was 42.36 ± 25.63%. Further patient characteristics are shown in Table 1 and the Supplementary Material.

**Table 1:** Characteristics of patients with CSDH included in the study.

Variable	No recurrence, *n* (%)/mean ± SD	Recurrence, *n* (%)/mean ± SD	Total, *n* (%)/mean ± SD	*p* value
Number (%)	221 (85.7)	37 (14.3)	258 (100)	
Sex, male	185 (83.7)	29 (78.4)	214 (82.9)	0.425
Age, years	69.88 ± 10.89	69.73 ± 10.72	69.86 ± 10.85	0.935
Age ≥80, years	37 (16.7)	8 (21.6)	45 (17.4)	0.469
BMI, kg/m^2^	22.36 ± 3.10	22.82 ± 2.81	22.43 ± 3.06	0.401
BMI ≥25.0, kg/m^2^	38 (17.2)	8 (21.6)	46 (17.8)	0.515
Bilateral surgery	27 (12.2)	8 (21.6)	35 (13.6)	0.122
Bilateral hematoma	59 (26.7)	11 (29.7)	70 (27.1)	0.701
Past medical history				
Hypertension	112 (50.7)	18 (48.6)	130 (50.4)	0.819
Diabetes	31 (14.0)	4 (10.8)	35 (13.6)	0.597
Heart disease	34 (15.4)	6 (16.2)	40 (15.5)	0.897
Antithrombotic therapy	28 (12.7)	5 (13.5)	33 (12.7)	0.887
Thrombocytopenia	10 (4.5)	3 (8.1)	13 (5.0)	0.408
Coagulation dysfunction	34 (15.4)	7 (18.9)	41 (15.9)	0.586
Laboratory investigation				
WBC, ×10^9^/L	6.67 ± 2.21	6.95 ± 1.87	6.71 ± 2.16	0.202
PLT, ×10^9^/L	188.65 ± 70.48	185.35 ± 65.40	188.18 ± 69.66	0.683
Eosinophils percentage	2.25 ± 2.21	1.42 ± 1.10	2.13 ± 2.11	**0** **.** **028**
Basophils percentage	0.52 ± 0.70	0.44 ± 0.19	0.51 ± 0.65	0.700
Eosinophils, ×10^9^/L	0.14 ± 0.14	0.09 ± 0.08	0.13 ± 0.13	0.055
Basophils, ×10^9^/L	0.02 ± 0.05	0.02 ± 0.02	0.02 ± 0.05	0.784
Radiological characteristics				
CT imaging of different types of CSDH				**0**.**012**
Gradation and separated types	34 (15.4)	12 (32.4)	46 (17.8)	
Homogeneous types and laminar or trabecular types	187 (84.6)	25 (67.6)	212 (82.2)	
Preoperative midline shift, mm	8.36 ± 4.32	8.87 ± 4.73	8.43 ± 4.38	0.439
PreHV, ml	119.10 ± 39.23	127.62 ± 47.37	120.32 ± 40.51	0.400
PostRCV, ml	65.94 ± 36.97	88.84 ± 42.46	69.22 ± 38.57	**<0** **.** **001**
PostRAV, ml	17.23 ± 21.44	27.86 ± 24.84	18.76 ± 22.23	**0** **.** **007**
PostRHV, ml	48.71 ± 25.07	60.97 ± 36.06	50.47 ± 27.18	**0** **.** **043**
Postoperative midline shift, mm	4.29 ± 3.26	5.24 ± 3.66	4.43 ± 3.33	0.110
Postoperative brain re-expansion rate, %	44.62 ± 24.69	28.85 ± 27.35	42.36 ± 25.63	**0** **.** **001**
CT imaging of different types of residual hematoma				**0**.**011**
Postoperative hybrid-density type	58 (26.2)	2 (8.1)	60 (23.3)	
Postoperative low-density type	121 (54.8)	23 (62.2)	144 (55.8)	
Postoperative high-density type	42 (19.0)	12 (32.4)	54 (20.9)	

n, number of patients; SD, standard deviation; BMI, body mass index; CSDH, chronic subdural hematoma; WBC, white blood cell count; PLT, platelet count; CT, computerized tomography; PreHV, preoperative hematoma volume; PostRCV, postoperative volume of the residual cavity; PostRAV, residual air volume; PostRHV, residual hematoma volume.

Boldface type indicates statistical significance.

The selection of cut-off values for continuous variables was based on the assessment of ROC curves to find the best predictive ability. Regarding the ROC curve of peripheral blood eosinophil count, the area under the ROC curve was 0.597 (95% CI 0.507–0.688, *p* = 0.058). A cut-off of peripheral blood eosinophil count at 0.15 × 10^9^/L produced a sensitivity of 83.8% and a specificity of 36.2%. Regarding the ROC curve of PreHV, the area under the ROC curve was 0.543 (95% CI 0.437–0.649, *p* = 0.400). A cut-off for PreHV at 155 ml produced a sensitivity of 32.4% and a specificity of 83.3%. Regarding the ROC curve of PostRCV, the area under the ROC curve was 0.681 (95% CI 0.591–0.771, *p* < 0.001). A cut-off for PostRCV at 70 ml produced a sensitivity of 70.3% and a specificity of 60.6%. The cut-off for PostRAV was 28 ml according to its ROC curve (AUC: 0.638, sensitivity: 48.6%, specificity: 78.3%). The cut-off for PostRHV was 55 ml (AUC: 0.604, sensitivity: 56.8%, specificity: 67.0%). Other cut-off values for continuous variables are shown in Table 2.

**Table 2:** Univariate and multivariate analyses of the association between CSDH recurrence and various variables.

Variable	Univariate analysis		Multivariate analysis	
	OR (95% CI)	*p* value	OR (95% CI)	*p* value
Sex, male	0.705 (0.298–1.667)	0.427		
Age ≥80, years	1.372 (0.581–3.238)	0.471		
BMI ≥25.0, kg/m^2^	1.328 (0.564–3.131)	0.516		
Bilateral surgery	1.982 (0.822–4.780)	0.128		
Bilateral hematoma	1.162 (0.540–2.497)	0.701		
Past medical history				
Hypertension	0.922 (0.459–1.850)	0.819		
Diabetes	0.743 (0.246–2.243)	0.598		
Heart disease	1.065 (0.413–2.746)	0.897		
Antithrombotic therapy	1.07 (0.387–2.994)	0.887		
Thrombocytopenia	1.862 (0.487–7.111)	0.363		
Coagulation dysfunction	1.283 (0.522–3.157)	0.587		
Laboratory investigation				
WBC (≥5.9 vs. <5.9) ×10^9^/L	2.058 (0.927–4.571)	0.076	1.666 (0.437–6.349)	0.455
PLT (≥187 vs. <187) ×10^9^/L	1.504 (0.748–3.026)	0.253		
Eosinophils percentage (<2.3 vs. ≥2.3)	3.354 (1.344–8.374)	**0** **.** **010**		
Basophils percentage (<0.75 vs. ≥0.75)	2.855 (0.653–12.476)	0.163		
Eosinophils (<0.15 vs. ≥0.15), ×10^9^/L	2.931 (1.173–7.328)	**0** **.** **021**	3.088 (1.158–8.231)	**0** **.** **024**
Basophils (≥0.03 vs. <0.03), ×10^9^/L	1.421 (0.680–2.968)	0.350		
Radiological characteristics				
Gradation and separated types (vs. other types)	2.640 (1.211–5.755)	**0** **.** **015**	3.137 (1.309–7.522)	**0** **.** **010**
Preoperative midline shift ≥9.5 mm (vs. <9.5 mm)	1.882 (0.934–3.795)	0.077	2.225 (1.017–4.867)	**0** **.** **045**
PreHV ≥155 ml (vs. <155 ml)	2.107 (0.978–4.539)	0.057		
PostRCV ≥70 ml (vs. <70 ml)	3.333 (1.591–6.985)	**0** **.** **001**	0.797 (0.201–3.157)	0.747
PostRAV ≥28 ml (vs. <28 ml)	3.325 (1.621–6.822)	**0** **.** **001**		
PostRHV ≥55 ml (vs. <55 ml)	2.385 (1.179–4.826)	**0** **.** **016**		
Postoperative midline shift ≥5.6 mm (vs. <5.6 mm)	2.132 (1.053–4.315)	**0** **.** **035**	1.257 (0.439–3.593)	0.670
Postoperative brain re-expansion rate <41% (vs. ≥41%)	3.464 (1.652–7.261)	**0** **.** **001**	3.958 (1.776–8.821)	**0** **.** **001**
CT imaging of different types of residual hematoma				
Hybrid-density type	0.161 (0.037–0.689)	**0** **.** **014**	0.173 (0.039–0.772)	**0** **.** **022**
Low-density type	5.512 (1.257–24.177)	**0** **.** **048**	4.786 (1.044–21.930)	**0** **.** **044**
Hyper-density type	8.286 (1.761–38.988)	**0** **.** **007**	9.591 (1.897–48.487)	**0** **.** **006**

OR, odds ratio; CI, confidence interval; BMI, body mass index; CSDH, chronic subdural hematoma; WBC, white blood cell count; PLT, platelet count; CT, computerized tomography; PreHV, preoperative hematoma volume; PostRCV, postoperative volume of the residual cavity; PostRAV, residual air volume; PostRHV, residual hematoma volume.

Boldface type indicates statistical significance.

Univariable logistic regression analyses demonstrated that the CSDH recurrence was significantly correlated with the percentage of eosinophils (OR 0.727, 95% CI 0.552–0.957, *p* = 0.023), peripheral blood eosinophil count <0.15 × 10^9^/L (OR 0.020, 95% CI 0.000–0.975, *p* = 0.049), gradation and separated types (OR 2.640, 95% CI 1.211–5.755, *p* = 0.015), PostRCV ≥70 ml (OR 3.333, 95% CI 1.591–6.985, *p* = 0.001), PostRAV ≥28 ml (OR 3.325, 95% CI 1.621–6.822, *p* = 0.001), PostRHV ≥55 ml (OR2.385, 95% CI 1.179–4.826, *p* = 0.016), residual hematoma width ≥1.4 cm (OR 3.093, 95% CI 1.429–6.695, *p* = 0.004), postoperative midline shift ≥5.6 mm (OR 2.132, 95% CI 1.053–4.315, *p* = 0.035), postoperative brain re-expansion rate <41% (OR 3.464, 95% CI 1.652–7.261, *p* = 0.001), postoperative hybrid-density type (OR, 0.161; 95% CI, 0.037–0.689; *p *= 0.014), postoperative low-density type (OR, 5.512; 95% CI, 1.257–24.177; *p *= 0.048), and postoperative high-density type (OR, 8.286; 95% CI, 1.761–38.988; *p *= 0.007) (Table 2 and Supplementary Material).

Collinearity diagnostics show that PreHV, PostRAV, PostRHV, and postoperative brain re-expansion rate are correlated, as well as eosinophils percentage and peripheral blood eosinophil count. Therefore, PreHV, PostRAV, PostRHV, and eosinophils percentage were excluded from the multivariable model. Other variables with a *p* value less than 0.10 in univariate analyses were included in the multivariate logistic regression (Table 2). In this multivariable model, significant independent predictors for recurrence of CSDH were as follows: peripheral blood eosinophil count <0.15 × 10^9^/L (OR, 3.024; 95% CI, 1.138–8.037; *p* = 0.026), gradation and separated types (OR, 3.163; 95% CI, 1.326–7.544; *p* = 0.009), preoperative midline shift ≥9.5 mm (OR, 2.216; 95% CI, 1.017–4.825; *p* = 0.045), postoperative brain re-expansion rate <41% (OR, 3.978; 95% CI, 1.792–8.830; *p *= 0.001), postoperative hybrid-density type (OR, 0.173; 95% CI, 0.039–0.772; *p* = 0.022), postoperative low-density type (OR, 4.786; 95% CI, 1.044–21.930; *p* = 0.044), and postoperative high-density type (OR, 9.591; 95% CI, 1.897–48.487; *p* = 0.006).

**Figure 3:**
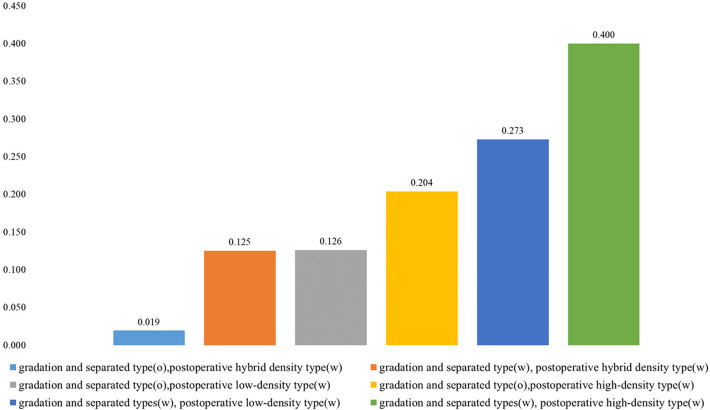
Recurrence rates (%) in patients with CSDH with or without gradation and separated types, postoperative hybrid-density type, postoperative low-density type, and/or postoperative high-density type.

To investigate the effect on recurrence due to postoperative hybrid-density type, postoperative low-density type, postoperative high-density type, gradation type, and separated type, we evaluated differences in the recurrence rate between patients with or without these factors (Figure 3). Patients were divided into six types according to CT imaging of different types of CSDH and PRH: (1) gradation and separated type-positive and postoperative high-density type-positive, (2) gradation and separated type-positive and postoperative low-density type-positive, (3) gradation and separated type-negative and postoperative high-density type-positive, (4) gradation and separated type-negative and postoperative low-density type-positive, (5) gradation and separated type-positive and postoperative hybrid-density-positive, and (6) gradation and separated type-negative and postoperative hybrid-density-positive. Recurrence rates were 40.0%, 27.3%, 20.4%,12.6%,12.5%, and 1.9%, respectively (*p* = 0.009). The recurrence rate in groups that were gradation type and separated type-positive and postoperative high-density type-positive was the highest among all groups, as shown in Figure 3.

There were no interactions between CT imaging of different types of CSDH and the percentage of eosinophils (*p* = 0.176), CT imaging of different types of CSDH and peripheral blood eosinophil count (*p* = 0.233), CT imaging of different types of PRH and the percentage of eosinophils (*p* = 0.654), or CT imaging of different types of PRH and peripheral blood eosinophil count (*p* = 0.329). The postoperative brain re-expansion rate was not associated with CT imaging of different types of PRH (*p* = 0.887). In patients with postoperative low-density type, the brain re-expansion rate was 28.72% ± 24.7% in the recurrence group and 45.90% ± 24.74% in the nonrecurrence group; these were significantly different (*p* = 0.003). In patients with postoperative high-density type, the brain re-expansion rate was 30.40% ± 34.62% in the recurrence group and 41.52% ± 29.90% in the nonrecurrence group; these were not significantly different (*p* = 0.371). Postoperative hybrid-density type appeared significantly more frequently in patients with the trabecular type (*p* = 0.016).

## Discussion

We observed an overall recurrence rate of 14.3% in patients with CSDH during a 3-month follow-up after burr-hole surgery. After adjusting for all relevant variables: peripheral blood eosinophil count <0.15 × 10^9^/L, gradation and separated types, preoperative midline shift ≥9.5 mm, postoperative brain re-expansion rate <41%, postoperative low-density type, and postoperative high-density type were significantly associated with CSDH recurrence in our study.

### Eosinophils

Eosinophils, cells of the innate immune system, derive from bone marrow and contain thick eosinophilic granules (23). In normal, the proportion of eosinophils in peripheral blood leukocytes is less than 5%, with a corresponding absolute eosinophil count of 0.5 × 10^9^/L (23). During host responses to helminth parasite infections or in allergic diseases such as asthma, eosinophil numbers in the blood and some tissues are known to increase (24). Therefore, peripheral blood eosinophil count has been incorporated into clinical prediction models by other studies. For example, peripheral blood eosinophil count is significantly associated with chronic rhinosinusitis in patients with chronic rhinitis (25). Peripheral blood eosinophil count less than 0.144 × 10^9^/L or 2% can predict a longer hospital length for patients who were admitted to the hospital with acute exacerbations of chronic obstructive pulmonary disease (26). CSDH is also an inflammatory and angiogenic disease associated with eosinophils (17). The presence of eosinophils within the outer membrane of CSDH has been confirmed in up to 50% of all CSDH surgical specimens (17, 18).

Unlike previous studies, the purpose of this study was to investigate the association between human peripheral blood eosinophils and the recurrence of CSDH after burr-hole surgery. In this study, peripheral blood eosinophil count was 0.13 ± 0.13 × 10^9^/L in total patients, accounting for 2.13% ± 2.11% of circulating leukocytes. The eosinophil count of the nonrecurrent group was significantly higher than that of the recurrent group (0.14 ± 0.14 × 10^9^/L vs. 0.09 ± 0.08 × 10^9^/L). Thus, our study suggested that eosinophils may play a protective role in preventing the recurrence of CSDH after burr-hole surgery. Similar conclusions have been drawn from a study about CSDH pathological specimens (21). Dense eosinophilic infiltration within the outer membrane was significantly associated with a lower risk of CSDH recurrence (21). They concluded that eosinophils may reduce the risk of recurrence of CSDHs by promoting membrane formation, repair, and fibrosis (21). This conclusion is also supported by our study on peripheral blood eosinophils.

The role of eosinophils in the life cycle of CSDH has remained controversial for a long time (17). Yamashima et al. suggested that eosinophils play an important role in maintaining the fluidity of the hematomas by secreting plasminogen-rich granules (19). In addition, Osuka et al. pointed out that transforming growth factor-beta, which is mainly secreted by eosinophils, can activate the fibrosis in CSDH outer membranes and contribute to the growth of CSDH (17). However, some researchers have put forward the opposite view that the infiltration of eosinophils may play a more important role in the repair and healing process of CSDH (18, 20). The frequency and extent of eosinophil infiltration increased with the duration of hematomas (18). In addition, according to the obvious relation of eosinophils with hemosiderin, some researchers proposed that eosinophils participate in the absorption of hematomas (18, 20). Thus, we think that eosinophils may play a dual role in the process of CSDH. In the development stage of CSDH, they may contribute to the growth of CSDH by activating the fibrosis of the outer membrane. As the hematoma matures, its impact on the absorption of the hematoma or fibrosis effect may play a beneficial role and reduce the risk of postoperative recurrence. This may be related to the different phenotypes of eosinophils in the process of inflammation.

### The classification of residual hematoma

Classification of CSDHs according to imaging appearance on CT scan has been widely used to predict the risk of postoperative recurrence (10, 16). However, the regularity of residual hematoma after burr-hole surgery has not been fully studied to date. Since all patients were irrigated with normal saline after the evacuation of the CSDH until the rinsing fluid became clean, we considered the fluid in the residual cavity after surgery to be normal saline (10). Therefore, we think that the residual hematoma should exhibit a homogeneous low density similar to that of cerebrospinal fluid on postoperative CT. This point was confirmed on CT imaging within 48–72 h of surgery in most patients (55.8%). However, there were still some patients who showed inhomogeneous density without and with high-density components on postoperative CT. We found that the group of low-density type and the group of high-density type had a 4.79- and 9.59-fold higher risk of CSDH recurrence than the group of hybrid-density type, respectively, after adjusting for all relevant variables.

**Figure 4:**
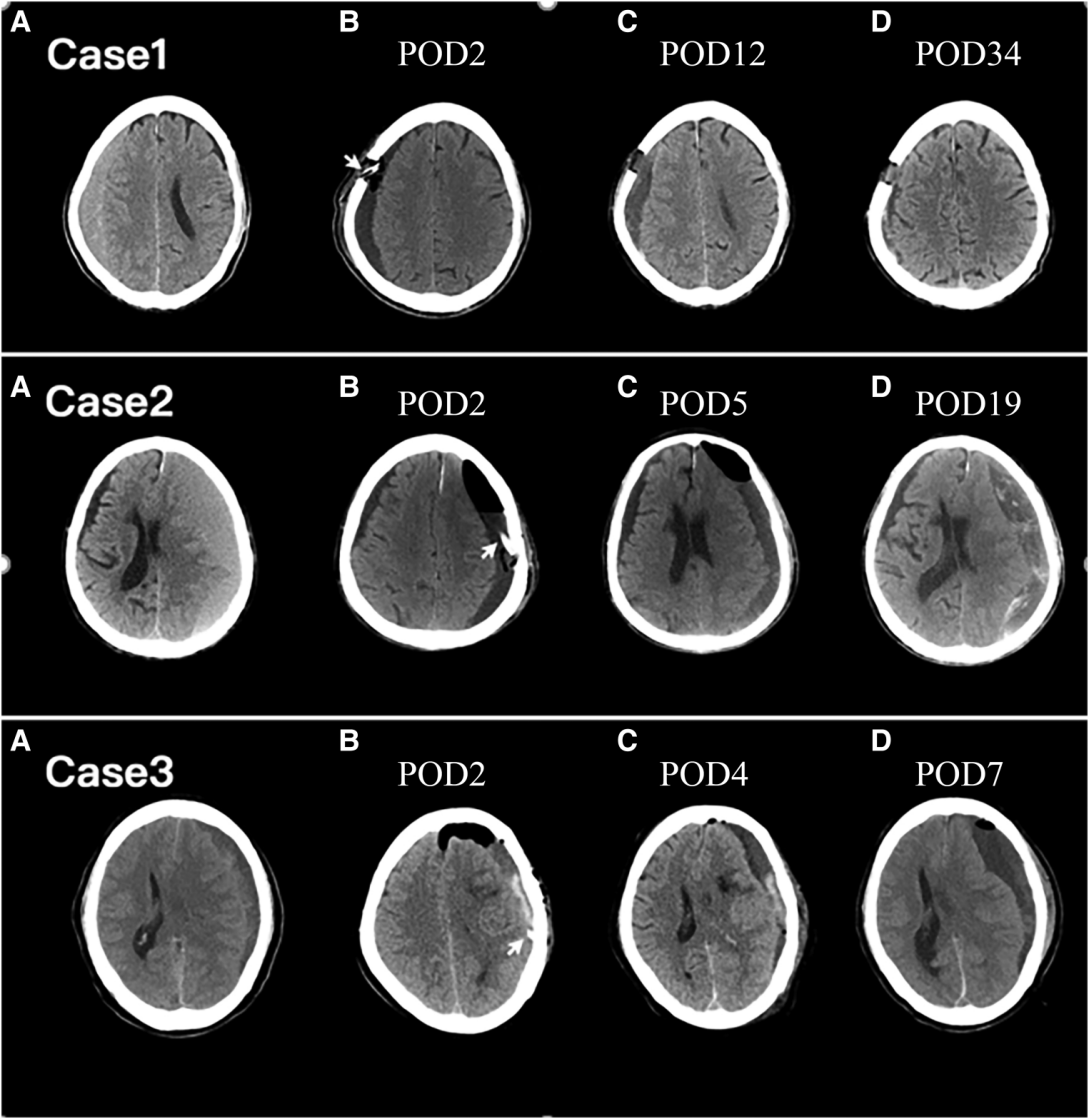
Axial CT images of representative cases. The arrowhead points to the drain tube. POD, postoperative day; Upper row: Case 1. Preoperative CT image (**A**) showing right-lateralized CSDH. Serial CT images were obtained on POD 2 (**B**), POD 12 (**C**), and POD 34 (**D**). A homogenous hypo-density type was exhibited on POD 2 (**B**). A hybrid-density type was exhibited at POD 12 (**C**) and the residual hematoma was almost completely reabsorption at POD 34 (**D**). Middle row: Case 2. Preoperative CT image (**A**) showing left-lateralized CSDH. Serial CT images were obtained on POD 2 (**B**), POD 5 (**C**), and POD 19 (**D**). A homogenous hypo-density type was exhibited on POD 2 (**B**). A hybrid-density type was exhibited at POD 5 (**C**). On POD 19, the residual hematoma was expanded and the recurrence happened (**D**). Lower row: Case 3. Preoperative CT image (**A**) showing left-lateralized CSDH. Serial CT images were obtained on POD 2 (**B**), POD 4 (**C**), and POD 7 (**D**). A layer of high CT density in the residual hematoma is demonstrated on POD 2 (**B**). On POD 4, the residual air was almost completely absorbed and the high-density component of the residual hematoma faded (**C**). CSDH recurrence occurred on POD 7 (**D**).

After checking the subsequent follow-up CT scans in the group of low-density type, we suggest that the resolution of residual hematoma can be divided into three stages: homogeneous low-density stage, heterogeneous hybrid-density stage, and resolution stage (Figure 4). During the homogeneous low-density stage, the residual hematoma is considered to be normal saline. As the residual hematoma began to be absorbed, fibrosis occurred and the density of the hematoma became hybrid. In the resolution stage, the residual hematoma was almost absorbed and the brain further rebounded. Therefore, the appearance of the hybrid-density type may indicate that the residual hematoma has entered the absorbed stage. In addition, we find that patients with the trabecular type of CSDH were more likely to enter the hybrid-density stage. This may be since trabecular CSDH contains a large number of septa created by fibrosis. This may explain that trabecular type have low risks for recurrence (15, 27).

However, not every patient in the group of low-density type eventually entered the resolution stage (Figure 4). In some patients, low-density residual hematoma lasted for a long time until the recurrence happened. The long-standing low-density residual hematoma may act as a subdural hygroma. The subdural hygroma is an accumulation of cerebrospinal fluid in the subdural space through a one-way valve caused by traumatic tearing or surgical dissection of the arachnoid membrane (28–30). Prolonged collection of cerebrospinal fluid in the subdural space triggers a complex inflammatory response, leading to the proliferation of dural border cells and the formation of the outer membrane of the hematoma (15, 31). Ultimately, microhemorrhages from neovascularization on the outer membrane of the hematoma lead to the formation of a CSDH (4, 32). The incidence of transformation from subdural hydroma to CSDH has been reported as varying from 4% to 58% in cases of past head injury (33). For patients after unruptured aneurysm surgery, the conversion rate from postoperative subdural hygroma to CSDH is reportedly as high as 27.1% (29). In our study, CSDH recurrence occurred in 16.0% of patients with the low-density type of PRH.

Although all patients had been irrigated with normal saline until the rinsing fluid became clean, the group of the high-density type still had active bleeding within 48–72 h of surgery (Figure 4). The high-density component of residual hematoma was regarded as the product of an acute subdural hematoma (ASDH) secondary to surgery. ASDH was put forward for the possible etiology for CSDH (4, 32). It is well recognized that 18%–21% of ASDH managed conservatively can transform into a CSDH over time (34, 35). Like ASDH, the most high-density component of residual hematoma was resorbed. However, the reabsorption process was incomplete in 22.2% of patients in the high-density group and contributed to the recurrence of CSDH. In conclusion, the lower risk of recurrence rate of the hybrid-density type is since the residual hematoma has entered the absorption stage, and the underlying mechanism may be that the hematoma is in an active process of fibrosis. The postoperative low-density type, acting as a subdural hydroma, has a higher risk of re-expansion, and the incomplete reabsorption process of ASDH contributes to the higher recurrence rate of the high-density type.

### Other variables

Radiological variables including preoperative hematoma width, preoperative midline shift, preoperative hematoma volume, postoperative residual air accumulation, CT imaging of different types of CSDH, and the residual total hematoma cavity volume have already been considered as predictive factors for CSDH recurrence (27, 36, 37). In the present study, gradation and separated types, PostRCV ≥70 ml, PostRAV ≥28 ml, PostRHV ≥55 ml, residual hematoma width ≥1.4 cm, postoperative midline shift ≥5.6 mm, and postoperative brain re-expansion rate <41% are risk factors of recurrence. However, only gradation and separated types, preoperative midline shift ≥9.5 mm, and postoperative brain re-expansion rate <41% are independent risk factors of recurrence, after adjusting for all relevant variables.

Nakaguchi et al. believed that CSDH develops as a homogeneous type initially, after which they sometimes develop in the laminar stage. Then, the hematoma gradually matures, which is represented by the separated stage. Finally, the hematoma eventually passes through the absorption stage as the trabecular type (15). Gradation type was included in the separated stage because the hematoma is not completely separated due to mild head movement (15). Compared with other hematoma types, the separated type of hematoma had significantly higher concentrations of fibrinogen, fibrin monomer, D dimer, interleukin-6, and interleukin-8, which indicates a higher tendency to rebleed, hyperfibrinolytic activity, and more severe inflammatory response (38). Therefore, the separated type of hematoma is active, with a higher postoperative recurrence rate. Our findings are consistent with those of other retrospective studies (15, 37, 38).

Previous studies showed that advanced age, brain atrophy, and compression by CSDH contribute to a higher brain-surface elastance (12, 39). A brain with high elastance helps predict poor re-expansion after the evacuation of the hematoma (39). Poor brain re-expansion implies the persistence of a subdural space, which creates a potential for the recurrence of CSDH (39, 40). In some studies, changes in hematoma thickness were used to define postoperative brain re-expansion rate, which was calculated as follows: (preoperative hematoma thickness − postoperative hematoma thickness)/preoperative hematoma thickness × 100 (%) (40, 41). In Mori's study, the poor re-expansion rate of the brain calculated by the change of hematoma thickness is significantly correlated to postoperative recurrence (40), but Miki et al. did not get significant results using the same way (41). We suggest that the re-expansion rate of the brain calculated by the change of hematoma thickness only used one layer of CT imaging, reflecting the limited re-expansion ability of the brain. Therefore, we propose to use the change in hematoma volume to calculate the re-expansion rate of the brain, as the difference between preoperative hematoma volume and postoperative residual cavity volume can directly reflect brain re-expansion ability. Larger PRH cavity volume has been previously reported significantly associated with the recurrence of CSDH (41, 42). It may prevent the re-expansion of the compressed brain; moreover, it may break the balance between reabsorption and microvascular leakage, leading to CSDH recurrence (42, 43). Residual air accumulation in the subdural may also prevent good re-expansion of the compressed brain (40). Preoperative midline shift was considered a predictive factor for CSDH recurrence in many studies (11, 36). This result may be attributed to the rapid expansion of the brain parenchyma after trephination, which exerts stress on the surrounding vessels and may contribute to acute rebleeding (11). A previous meta-analysis reported that antithrombotic drugs increased the risk of reoperation rate in patients with CSDH (44). However, this study shows that antithrombotic use is not associated with CSDH recurrence. There are also some retrospective studies that have yielded similar results (10, 36, 45, 46). Preoperative antithrombotic use does not seem to influence the rate of CSDH recurrence and early antithrombotic recommencement may reduce the risk of thromboembolic events (45, 46).

## Limitations

There were several limitations recognized in this study. First, this study is a retrospective study and included a relatively small sample. Due to the retrospective nature of the study, the findings were potentially subject to sources of bias and variation. Second, the study was conducted at a single center, which may impact the generalizability of the results, and thus external validation from data collected at other centers might be required. Third, considering that the role eosinophils play in the CSDH recurrence is still confusing, more studies need to further and better delineate the mechanism underlying the association of eosinophils with the recurrence of CSDH. The correlation between peripheral blood eosinophils and eosinophilic infiltration of the CSDH outer membrane also requires further investigation. In addition, we were unable to accurately evaluate how long it would take for the homogeneous low-density residual hematoma to enter the hybrid-density stage due to irregular and rare postoperative CT scans. Therefore, more studies need to focus on the absorption process of homogeneous low-density residual hematoma. Although postoperative low-density type and postoperative high-density type were identified as risk factors for postoperative CSDH recurrence in this study, large confidence intervals for both factors support a certain uncertainty in these results.

## Conclusion

The lower peripheral blood eosinophil count, gradation and separated types, higher preoperative midline shift, lower postoperative brain re-expansion rate, postoperative low-density type, and postoperative high-density type are significant independent predictors for CSDH recurrence after burr-hole surgery. Eosinophils may reduce the risk of postoperative recurrence by exerting their fibrotic effects. We suggest that the resolution of residual hematoma can be divided into three stages: homogeneous low-density stage, heterogeneous hybrid-density stage, and resolution stage. The long-standing low-density residual hematoma may act as a subdural hydroma with a higher risk of re-expansion. However, the appearance of hybrid-density type within 48–72 h of surgery may indicate that the residual hematoma has entered the absorbed stage with a lower recurrence rate. The incomplete reabsorption process of ASDH contributes to the higher recurrence rate of the high-density type.

## Data Availability

The raw data supporting the conclusions of this article will be made available by the authors, without undue reservation.

## References

[1] Yang W Huang J. Chronic subdural hematoma: epidemiology and natural history. Neurosurg Clin N Am. (2017) 28(2):205–10. 10.1016/j.nec.2016.11.002 28325454

[2] Rauhala M Luoto TM Huhtala H Iverson GL Niskakangas T Ohman J The incidence of chronic subdural hematomas from 1990 to 2015 in a defined Finnish population. J Neurosurg. (2019) 132(4):1147–57. 10.3171/2018.12.JNS183035 30901751

[3] Hirai S Yagi K Hara K Kanda E Matsubara S Uno M. Postoperative recurrence of chronic subdural hematoma is more frequent in patients with blood type A. J Neurosurg. (2021) 135(4):1203–7. 10.3171/2020.7.Jns202330 33385994

[4] Kolias AG Chari A Santarius T Hutchinson PJ. Chronic subdural haematoma: modern management and emerging therapies. Nat Rev Neurol. (2014) 10(10):570–8. 10.1038/nrneurol.2014.163 25224156

[5] Ivamoto HS Lemos HP Jr., Atallah AN. Surgical treatments for chronic subdural hematomas: a comprehensive systematic review. World Neurosurg. (2016) 86:399–418. 10.1016/j.wneu.2015.10.025 26485412

[6] Yu GJ Han CZ Zhang M Zhuang HT Jiang YG. Prolonged drainage reduces the recurrence of chronic subdural hematoma. Br J Neurosurg. (2009) 23(6):606–11. 10.3109/02688690903386983 19922274

[7] Cofano F Pesce A Vercelli G Mammi M Massara A Minardi M Risk of recurrence of chronic subdural hematomas after surgery: a multicenter observational cohort study. Front Neurol. (2020) 11:560269. 10.3389/fneur.2020.560269 33329304 PMC7732444

[8] Chari A Kolias AG Santarius T Bond S Hutchinson PJ. Twist-drill craniostomy with hollow screws for evacuation of chronic subdural hematoma. J Neurosurg. (2014) 121(1):176–83. 10.3171/2014.4.JNS131212 24785319

[9] Santarius T Kirkpatrick PJ Kolias AG Hutchinson PJ. Working toward rational and evidence-based treatment of chronic subdural hematoma. Clin Neurosurg. (2010) 57:112–22. 21280503

[10] Stanisic M Pripp AH. A reliable grading system for prediction of chronic subdural hematoma recurrence requiring reoperation after initial burr-hole surgery. Neurosurgery. (2017) 81(5):752–60. 10.1093/neuros/nyx090 28379528 PMC5808673

[11] Schwarz F Loos F Dunisch P Sakr Y Safatli DA Kalff R Risk factors for reoperation after initial burr hole trephination in chronic subdural hematomas. Clin Neurol Neurosurg. (2015) 138:66–71. 10.1016/j.clineuro.2015.08.002 26282910

[12] Han MH Ryu JI Kim CH Kim JM Cheong JH Yi HJ. Predictive factors for recurrence and clinical outcomes in patients with chronic subdural hematoma. J Neurosurg. (2017) 127(5):1117–25. 10.3171/2016.8.Jns16867 27982768

[13] Santarius T Kirkpatrick P Ganesan D Chia H Jalloh I Smielewski P Use of drains versus no drains after burr-hole evacuation of chronic subdural haematoma: a randomised controlled trial. Lancet. (2009) 374(9695):1067–73. 10.1016/s0140-6736(09)61115-6 19782872

[14] Motoie R Karashima S Otsuji R Ren N Nagaoka S Maeda K Recurrence in 787 patients with chronic subdural hematoma: retrospective cohort investigation of associated factors including direct oral anticoagulant use. World Neurosurg. (2018) 118:e87–91. 10.1016/j.wneu.2018.06.124 29945004

[15] Nakaguchi H Tanishima T Yoshimasu N. Factors in the natural history of chronic subdural hematomas that influence their postoperative recurrence. J Neurosurg. (2001) 95(2):256–62. 10.3171/jns.2001.95.2.0256 11780895

[16] Won SY Dubinski D Eibach M Gessler F Herrmann E Keil F External validation and modification of the Oslo grading system for prediction of postoperative recurrence of chronic subdural hematoma. Neurosurg Rev. (2021) 44(2):961–70. 10.1007/s10143-020-01271-w 32112162

[17] Osuka K Watanabe Y Usuda N Aoyama M Takeuchi M Takayasu M. Eotaxin-3 activates the smad pathway through the transforming growth factor beta 1 in chronic subdural hematoma outer membranes. J Neurotrauma. (2014) 31(16):1451–6. 10.1089/neu.2013.3195 24684589

[18] Sarkar C Lakhtakia R Gill SS Sharma MC Mahapatra AK Mehta VS. Chronic subdural haematoma and the enigmatic eosinophil. Acta Neurochir (Wien). (2002) 144(10):983–8, discussion 8. 10.1007/s00701-002-0994-6 12382126

[19] Yamashima T Kubota T Yamamoto S. Eosinophil degranulation in the capsule of chronic subdural hematomas. J Neurosurg. (1985) 62(2):257–60. 10.3171/jns.1985.62.2.0257 3968565

[20] Muller W Firsching R. Significance of eosinophilic granulocytes in chronic subdural hematomas. Neurosurg Rev. (1990) 13(4):305–8. 10.1007/BF00346370 2280841

[21] Davidson B Narvacan K Munoz DG Rotondo F Kovacs K Zhang S The crucial role of eosinophils in the life cycle, radiographical architecture, and risk of recurrence of chronic subdural hematomas. Neurotrauma Rep. (2021) 2(1):76–83. 10.1089/neur.2020.0036 34223547 PMC8240825

[22] Sucu HK Gokmen M Gelal F. The value of XYZ/2 technique compared with computer-assisted volumetric analysis to estimate the volume of chronic subdural hematoma. Stroke. (2005) 36(5):998–1000. 10.1161/01.STR.0000162714.46038.0f 15817899

[23] Weller PF Spencer LA. Functions of tissue-resident eosinophils. Nat Rev Immunol. (2017) 17(12):746–60. 10.1038/nri.2017.95 28891557 PMC5783317

[24] Huang L Gebreselassie NG Gagliardo LF Ruyechan MC Lee NA Lee JJ Eosinophil-derived IL-10 supports chronic nematode infection. J Immunol. (2014) 193(8):4178–87. 10.4049/jimmunol.1400852 25210122 PMC4241261

[25] Oh SY Hwang J Ryu YJ Won JY Kwon SO Lee WH. Blood eosinophils may predict radiographic sinus opacification in patients with chronic rhinitis. Int Forum Allergy Rhinol. (2019) 9(5):522–7. 10.1002/alr.22270 30576087

[26] Ko FWS Chan KP Ngai J Ng SS Yip WH Ip A Blood eosinophil count as a predictor of hospital length of stay in COPD exacerbations. Respirology. (2020) 25(3):259–66. 10.1111/resp.13660 31385389

[27] Yamamoto H Hirashima Y Hamada H Hayashi N Origasa H Endo S. Independent predictors of recurrence of chronic subdural hematoma: results of multivariate analysis performed using a logistic regression model. J Neurosurg. (2003) 98(6):1217–21. 10.3171/jns.2003.98.6.1217 12816267

[28] Lee KS. The pathogenesis and clinical significance of traumatic subdural hygroma. Brain Inj. (1998) 12(7):595–603. 10.1080/026990598122359 9653522

[29] Park J Cho JH Goh DH Kang DH Shin IH Hamm IS. Postoperative subdural hygroma and chronic subdural hematoma after unruptured aneurysm surgery: age, sex, and aneurysm location as independent risk factors. J Neurosurg. (2016) 124(2):310–7. 10.3171/2015.1.JNS14309 26275003

[30] Yoshimoto Y Wakai S Hamano M. External hydrocephalus after aneurysm surgery: paradoxical response to ventricular shunting. J Neurosurg. (1998) 88(3):485–9. 10.3171/jns.1998.88.3.0485 9488302

[31] Takahashi Y Mikami J Ueda M Ito K Sato H Matsuoka T The origin of chronic subdural hematoma considered on the basis of hematoma membrane findings and contained fluid findings. Neurol Med Chir. (1985) 25(12):998–1009. 10.2176/nmc.25.998 2422579

[32] Lee KS. Natural history of chronic subdural haematoma. Brain Inj. (2004) 18(4):351–8. 10.1080/02699050310001645801 14742149

[33] Lee KS Bae WK Bae HG Yun IG. The fate of traumatic subdural hygroma in serial computed tomographic scans. J Korean Med Sci. (2000) 15(5):560–8. 10.3346/jkms.2000.15.5.560 11068995 PMC3054688

[34] Edlmann E Whitfield PC Kolias A Hutchinson PJ. Pathogenesis of chronic subdural hematoma: a cohort evidencing de novo and transformational origins. J Neurotrauma. (2021) 38(18):2580–9. 10.1089/neu.2020.7574 33787358

[35] Izumihara A Yamashita K Murakami T. Acute subdural hematoma requiring surgery in the subacute or chronic stage. Neurol Med Chir. (2013) 53(5):323–8. 10.2176/nmc.53.323 23708224

[36] Ha BJ Bae IS Kim JM Cheong JH Ryu JI Han MH. Effects of possible osteoporotic conditions on the recurrence of chronic subdural hematoma. Front Neurol. (2020) 11:538257. 10.3389/fneur.2020.538257 33071940 PMC7542308

[37] Chon KH Lee JM Koh EJ Choi HY. Independent predictors for recurrence of chronic subdural hematoma. Acta Neurochir (Wien). (2012) 154(9):1541–8. 10.1007/s00701-012-1399-9 22653496

[38] Nomura S Kashiwagi S Fujisawa H Ito H Nakamura K. Characterization of local hyperfibrinolysis in chronic subdural hematomas by SDS-PAGE and immunoblot. J Neurosurg. (1994) 81(6):910–3. 10.3171/jns.1994.81.6.0910 7965121

[39] Fukuhara T Gotoh M Asari S Ohmoto T Akioka T. The relationship between brain surface elastance and brain reexpansion after evacuation of chronic subdural hematoma. Surg Neurol. (1996) 45(6):570–4. 10.1016/0090-3019(95)00471-8 8638244

[40] Mori K Maeda M. Surgical treatment of chronic subdural hematoma in 500 consecutive cases: clinical characteristics, surgical outcome, complications, and recurrence rate. Neurol Med Chir. (2001) 41(8):371–81. 10.2176/nmc.41.371 11561347

[41] Miki K Abe H Morishita T Hayashi S Yagi K Arima H Double-crescent sign as a predictor of chronic subdural hematoma recurrence following burr-hole surgery. J Neurosurg. (2019) 131(6):1905–11. 10.3171/2018.8.Jns18805 30611142

[42] Jack A O'Kelly C McDougall C Findlay JM. Predicting recurrence after chronic subdural haematoma drainage. Can J Neurol Sci. (2015) 42(1):34–9. 10.1017/cjn.2014.122 25557536

[43] Markwalder TM. Chronic subdural hematomas: a review. J Neurosurg. (1981) 54(5):637–45. 10.3171/jns.1981.54.5.0637 7014792

[44] Wang H Zhang M Zheng H Xia X Luo K Guo F The effects of antithrombotic drugs on the recurrence and mortality in patients with chronic subdural hematoma: a meta-analysis. Medicine. (2019) 98(1):e13972. 10.1097/MD.0000000000013972 30608437 PMC6344112

[45] Aspegren OP Astrand R Lundgren MI Romner B. Anticoagulation therapy a risk factor for the development of chronic subdural hematoma. Clin Neurol Neurosurg. (2013) 115(7):981–4. 10.1016/j.clineuro.2012.10.008 23128014

[46] Poon MTC Rea C Kolias AG Brennan PM. British neurosurgical trainee research C. Influence of antiplatelet and anticoagulant drug use on outcomes after chronic subdural hematoma drainage. J Neurotrauma. (2021) 38(8):1177–84. 10.1089/neu.2018.6080 30526281 PMC8060161

