# Supplementary material for: Peripheral blood eosinophil and classification of residual hematoma help predict the recurrence of chronic subdural hematoma after initial surgery
Source: Front Surg. 2022 Sep 23;9:970468. doi: 10.3389/fsurg.2022.970468 (PMC9632999; doi:10.3389/fsurg.2022.970468)
Supplement: Supplementary file 1 [file Table1.docx]

Supplementary Material

# Supplementary Table

**TABLE 1 Continued. Characteristics of patients with CSDH included in the study**

| Variable | No Recurrence  n (%)/mean ± SD | Recurrence  n (%)/mean ± SD | Total  n (%)/mean ± SD | P Value |
| --- | --- | --- | --- | --- |
| **Past medical history** | | | | |
| Chronic kidney disease | 4(1.8) | 0(0) | 4(1.6) | 1.000 |
| Neurosurgery history | 11(5.0) | 2(5.4) | 13(5.0) | 1.000 |
| Spontaneous intracranial hypotension | 7(3.2) | 2(5.4) | 9(3.5) | 0.621 |
| Preoperative GCS score:15 | 185(83.7) | 31(83.8) | 216(83.7) | 0.991 |
| Speech deficiency related to CSDH | 28(12.7) | 4(10.8) | 32(12.4) | 0.751 |
| **Laboratory investigation** | | | | |
| Neutrophils percentage, % | 67.32±10.25 | 67.37±9.07 | 67.33±10.07 | 0.979 |
| Neutrophil, ×10^9^/L | 5.17±9.02 | 4.71±1.60 | 5.10±8.37 | 0.342 |
| **Radiological characteristics** | | | | |
| Preoperative hematoma width, mm | 2.27±0.92 | 2.37±0.83 | 2.28±0.91 | 0.442 |
| Residual hematoma width, mm | 1.50±0.97 | 1.80±0.66 | 1.54±0.93 | **0.004** |

n, number of patients; SD, standard deviation; GCS, Glasgowcoma scale; CSDH, chronic subdural hematoma;

Boldface type indicates statistical significance.

**TABLE 2 Continued. Univariate and multivariate analyses of the** **association between CSDH recurrence and various variables**

| Variable | Univariate Analysis | | Multivariate Analysis | |
| --- | --- | --- | --- | --- |
|  | OR (95% CI) | P Value | OR (95% CI) | P Value |
| Chronic kidney disease | 0.000(0.000) | 0.999 |  |  |
| Neurosurgery History | 1.091(0.232-5.132) | 0.912 |  |  |
| Spontaneous intracranial hypotension | 1.747(0.349-8.753) | 0.497 |  |  |
| Preoperative GCS score:15 | 0.995(0.387-2.557) | 0.991 |  |  |
| Speech deficiency related to CSDH | 0.835(0.275-2.537) | 0.751 |  |  |
| **Laboratory investigation** | | | | |
| Neutrophils percentage (≥61vs<61) % | 1.478(0.641-3.406) | 0.359 |  |  |
| Neutrophil (≥4.1vs<4.1), ×10^9^/L | 1.955(0.936-4.087) | 0.075 | 0.943(0.276-3.225) | 0.925 |
| **Radiological characteristics** | | | | |
| Preoperative hematoma width≥2.2cm (vs<2.2cm) | 1.424(0.706-2.873) | 0.324 |  |  |
| Residual hematoma width≥1.4cm(vs<1.4cm) | 3.093(1.429-6.695) | **0.004** | 1.330(0.433-4.092) | 0.618 |

OR, odds ratio; CI, confidence interval; GCS, Glasgowcoma scale; CSDH, chronic subdural hematoma;

Boldface type indicates statistical significance.
